# Control of Plant Viruses by CRISPR/Cas System-Mediated Adaptive Immunity

**DOI:** 10.3389/fmicb.2020.593700

**Published:** 2020-10-26

**Authors:** Yongsen Cao, Huanbin Zhou, Xueping Zhou, Fangfang Li

**Affiliations:** ^1^State Key Laboratory for Biology of Plant Diseases and Insect Pests, Institute of Plant Protection, Chinese Academy of Agricultural Sciences, Beijing, China; ^2^State Key Laboratory of Rice Biology, Institute of Biotechnology, Zhejiang University, Hangzhou, Zhejiang, China

**Keywords:** CRISPR, Cas proteins, plant virus, recessive resistance genes, antiviral engineering

## Abstract

Plant diseases caused by invading plant viruses pose serious threats to agricultural production in the world, and the antiviral engineering initiated by molecular biotechnology has been an effective strategy to prevent and control plant viruses. Recent advances in clustered regularly interspaced short palindromic repeats (CRISPR)/CRISPR-associated (Cas) system-mediated DNA or RNA editing/interference in plants make them very attractive tools applicable to the plant protection field. Here, we review the development of CRISPR/Cas systems and summarize their applications in controlling different plant viruses by targeting viral sequences or host susceptibility genes. We list some potential recessive resistance genes that can be utilized in antiviral breeding and emphasize the importance and promise of recessive resistance gene-based antiviral breeding to generate transgene-free plants without developmental defects. Finally, we discuss the challenges and opportunities for the application of CRISPR/Cas techniques in the prevention and control of plant viruses in the field.

## Introduction

Plant diseases caused by plant pathogens, including bacteria, fungi, nematodes, and viruses in different crops, lead to enormous economic losses worldwide. Plant viruses cause almost half of plant diseases, and the annual global cost of viral infection of cultivated crops is estimated to be more than US$ 30 billion (Nicaise, 2014; Sastry and Zitter, 2014). About 1,500 plant virus species, grouped into 26 families, have been identified and characterized based on viral genome sequences so far^1^. Plant viruses are a class of obligate intracellular parasites, with minimal coding capacity, which heavily rely on their host to complete their infection/life cycle. Unlike in the case of other pathogens, there are barely any efficient antibiotics or other chemicals that can eliminate an infecting plant virus without perturbing host cells. Therefore, molecular plant breeding plays a pivotal role in generating virus immunity, virus resistance, or virus-tolerant plants in agricultural production to prevent and control plant viruses.

**FIGURE 1:**
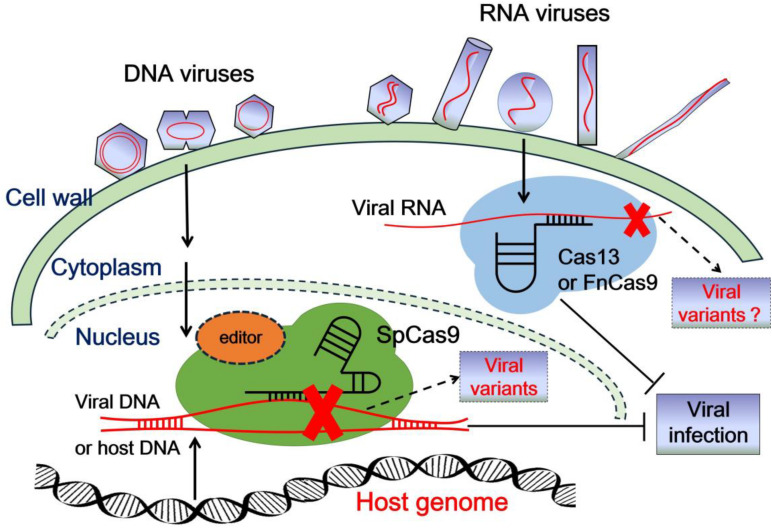
Schematic representation of class 2 CRISPR/Cas systems to confer resistance to plant viruses. After plant viruses enter into plant cells, the viral genome is uncoated and transcribed, or translated with the help of host factors. Plant viruses then multiply their genome in the nucleus or in the cytoplasm. The viral genome DNA or RNA can be targeted, destroyed, or interfered with by CRISPR/Cas9 or CRISPR/Cas13 (or FnCas9) systems in the nucleus or in the cytoplasm, respectively, which inhibits viral infection. In addition, the mutation or deficiency in host susceptibility factors edited by the CRISPR/Cas9 system also perturbs viral infection. However, some new viral variants might be generated as by-products when CRISPR/Cas systems edit the viral genome, which might drive the risk of viral evolution.

CRISPR (clustered regularly interspaced short palindromic repeats)/CRISPR-associated (Cas) systems, derived from bacterial and archaeal sources, serve as an important adaptive immunity against invading nucleic acids. With the fast development of our understanding of CRISPR/Cas systems, they have been converted into convenient tools to edit endogenous and exogenous DNA or RNA sequences in different organisms. To date, a simplified CRISPR system only contains an easily engineered guide RNA (gRNA) and a Cas effector protein. Based on the different characteristics of Cas proteins in sequence, structure, and function, CRISPR/Cas systems are separated into two distinct classes (Makarova et al., 2015, 2017a,b). Class 1 of CRISPR/Cas systems contains types I, III, and IV, which utilize a multi-protein effector complex (Makarova et al., 2017a). In contrast, class 2 of CRISPR/Cas systems includes types II, V, and VI, which achieve target editing only with a single effector protein (Makarova et al., 2017b; Shmakov et al., 2017). Therefore, class 2 CRISPR/Cas systems have been widely adopted and utilized for nucleic acid manipulation and detection (Hsu et al., 2013; Garcia-Doval and Jinek, 2017; Ji et al., 2019; Table 1). Among them, the type II and V Cas proteins are utilized to edit DNA, and the type VI Cas proteins are applied for editing RNA. In the past few years, CRISPR/Cas system-mediated gene editing technology has been introduced rapidly into plant genetic engineering to generate resistance against viruses and other pathogens (Hadidi et al., 2016; Ma et al., 2016; Langner et al., 2018; Mahas and Mahfouz, 2018; Chen et al., 2019; Li and Wang, 2019; Li et al., 2019; Zhang et al., 2019b). Generally, there are two main strategies to utilize CRISPR/Cas technology to control plant viruses (Figure 1). One approach is to target, destroy, and interfere with the viral genome to inhibit the replication and infection of invading viruses. The other one is to manipulate host susceptibility factors required for the viral infection/life cycle in order to enhance plant immunity and block virus invasion.

**TABLE 1:** CRISPR/Cas system-mediated resistance to plant viruses by targeting the viral genome, host factors, and some potential recessive targets using genome editing systems.

**Targets**	**Viruses**	**Applied systems**	**Potential recessive resistance targets (Without development defects)**	**Viruses**
Viral replication region	TYLCCNV, TbCSV	ZFN	AOCl,AT4CLI,BAMI,CML38,CML39,	Geminiviruses
Viral IR region/replication region	TYLCCNV, TbCSV	TALEN	CPHSC70-1, CSNs, deltaCOP, GRAB2, HSC70, NSI, NsAK, PLP2, rgs-CaM, SCE1, SK4-I/SKK, SYTA1	
Vil-Hl IR region/replication region	BeYDV. CLCuMuV	CRlSPR/SpCas9		
Viral IR region/replication region/CP	BCTV, CLC’uKoV, McMV, TYLCV	CRJSPR/SpCas9	u-EXPA, bZIP60, CK2, cPGK, DBP1, DDXs, DnaJ-like proteins, eEFl A, eEFIB, eEF4E, eEF4G, eEF4G2, eEF(iso)4E, GRF6, IREI A, IRE1B, NBR1, PABPs, PCaPl, PVIPs, RAV2, RHs (RH8, RH9), Sec24a, SYTA1, SYP71	Potyviruses
Viral genome region	BSCTV	C’RISPR/SpCas9	l > ZIP60, CAT1, cPGK, eEFIB, EXA1	Potexviruses
Viral long IR region/MP/CP/replication region	WDV, BSV	CRlSPR/SpCas9	ARL8a/8b, eEFIA, SYTA1, TOM1, TOM2A, TOM3	Tobamoviruses
Viral CP	CaMV	CRISPR/SpCas9	Chl-PCK, eEFIA	BaMV
Viral coding ORF/non-coding region/3′ UTR	TMV, CMV	CRISPR/FnCas9	PDIL5-1	BaYMV, BaMMV
Viral HC-Pro/CP/GFP region	TuMV (TuMV-GFP)	CRISPR/LshCasl3a, Rfi(Casl3d	FDH, CRT3, HAT1, HAT2, HAT3, VPS41	CMV
Viral coding ORF	TMV, SRBSDV	(RISPR LshCa.sl3a	PDLP1/PDLP2/PDLP3	GFLV.CaMV
Viral NIh/P3/CI/CP region	PVY	C’RISPR/LshCasl3a	EXAI	LoLV
Host gene elF4E	CVYV, ZYMV, PRSV-W, TuMV	CRISPR’SpCas9	eEFIA, ESCRT, GAPDH, RAB5, SYPK1	TBSV
Host gene elF4E	C1YVV	CRISPR/nCaaS- cytidine deaminase	SYTA1	TVCV
Host gene el F4G	RTSV	CRISPR/SpCas9	CPR5	RYMV
Host gene elF(iso)4E	TuMV	CRISPR/SpCas9	RIM1.SAMS1	RDV
Host gene nCBP-l/nCBP-2	CBSV	CRISPR/SpCas9	RHD3	TSWV

*The known targets in *light blue background* are used in antiviral engineering by genome editing technologies, which can be viral replication regions, viral open reading frames (ORFs), viral non-coding regions, or the host factors required for viral infection. The potential recessive targets in *light yellow background* which are suppressors of plant defenses, the susceptibility factors required for viral translation/replication/movement or other life cycles, the factors in the cellular metabolism, or the modified factors for the benefit of the viral infection or viral protein functions, etc., can be utilized in antiviral engineering by genome editing technologies. The abbreviations of the virus names in this table are as follows: *BaMMV*, barley mild mosaic virus; *BaYMV*, barley yellow mosaic virus. Caulimoviruses: *BSV*, banana streak virus; *CaMV*, cauliflower mosaic virus; *CMV*, cucumber mosaic virus; *CMV-GFP*, cucumber mosaic virus carrying GFP. Geminiviruses: *BCTV*, beet curly top virus; *BeYDV*, bean yellow dwarf virus; *BSCTV*, beet severe curly top virus; *CLCuKoV*, cotton leaf curl Kokhran virus; *CLCuMuV*, cotton leaf curl Multan virus; *MeMV*, Merremia mosaic virus; *TbCSV*, tobacco curly shoot virus; *TYLCCNV*, tomato yellow leaf curl China virus; *TYLCV*, tomato yellow leaf curl virus; *TYLCSV*, tomato yellow leaf curl Sardinia virus; *WDV*, wheat dwarf virus; *GFLV*, grapevine fan leaf virus; *LoLV*, Lolium latent virus. Potyviruses: *CBSV*, cassava brown streak virus; *ClYVV*, clover yellow vein virus; *CVYV*, cucumber vein yellowing virus; *PRSV-W*, papaya ringspot virus-W; *PVY*, potato virus Y; *TuMV*, turnip mosaic virus; *TuMV-GFP*, turnip mosaic virus carrying GFP; *ZYMV*, zucchini yellow mosaic virus; *TBSV*, tomato bushy stunt virus; *RDV*, rice dwarf virus; *RTSV*, rice tungro spherical virus; *RYMV*, rice yellow mottle virus; *SRBSDV*, southern rice black-streaked dwarf virus; *TMV*, tobacco mosaic virus; *TMV-GFP*, tobacco mosaic virus carrying GFP; *TSWV*, tomato spotted wilt virus. The abbreviations of the gene names in this table are listed as follows. Potential recessive resistance targets that function in the infection of geminiviruses: *AOC1*, allene oxide cyclase 1; *AT4CL1*, 4-coumarate: CoA ligase; *BAM1*, barely any meristem 1; *CML38*/*39*, calmodulin-like protein 38/39; *CPHSC70-1*, chloroplastic HSC70-1; *CSNs*, COP9 signalosome subunits; *delta COP*, coatomer delta subunit; *GRAB2*, geminivirus Rep A-binding; *HSC70*, heat shock protein cognate; *NSI*, nuclear acetyltransferase I; *NsAK*, a proline-rich extensin-like receptor protein kinase (PERK); *PLP2*, patatin-like protein 2; *rgs-CaM*, regulator of RNA silencing, calmodulin-like protein; SCE1, *SUMO*, conjugating enzyme E1; *SK4-1/SKK*, shaggy-related kinase proteins. Potential recessive resistance targets that function in the infection of potyviruses: *α-EXPA1*, expansin; *bZIP60*, basic-leucine zipper transcription factor 60; *cPGK*, nuclear-encoded chloroplast phosphoglycerate kinase; *DDXs* or *RHs*, DEAD-box RNA helicase; *eEF1*, eukaryotic translation elongation factor 1A/1B; *eIF4s/eIF(iso)4s*, eukaryotic translation initiation factors 4/isoform factors 4 [eIF4E, eIF4G, eIF4G2, eIF(iso)4E, etc.]; *GRF6*, 4-3-3 family protein; *IRE1*, inositol requiring protein-1; *NBR1*, neighbor of BRCA1 gene 1; *PABPs*, polyA-binding proteins; *PCaP1*, plasma membrane-associated cation-binding protein-1; *PVIPs*, potyvirus VPg-interacting proteins; *RAV2*, ethylene-inducible host transcription factor; *Sec24a*, a COPII coatomer; *SYTA1*, synaptotagmin 1; *Syp71*, snare protein 71. Potential recessive resistance targets that function in the infection of CMV: *FDH*, formate dehydrogenase; *CRT3*, calreticulin-3; *HATs*, homeodomain-leucine zipper proteins 1/2/3; *VPS41*, vacuolar protein sorting 41. Potential recessive resistance targets that function in the infection of potexviruses: bZIP60; *CAT1*, catalase 1; cPGK; *EXA1*, essential for poteXvirus accumulation 1, including a putative eIF4E-binding motif and a GYF domain protein. Potential recessive resistance targets that function in the infection of tobamoviruses: *ARL8a/8b*, a small GTP-binding ARF family protein 8a/8b; *TOM*, tobamovirus multiplication (TOM1, TOM2A, and TOM3). Potential recessive resistance targets that function in the infection of some other viruses: *chl-PGK*, chloroplast phosphoglycerate kinase; *CPR5*, also referred to as the RYMV2 gene; *ESCRT*, endosomal sorting complexes required for transport; *GAPDH*, glyceraldehyde 3-phosphate dehydrogenase; *PDIL5-1*, protein disulfide isomerase-like 5-1; *PDLP1/2/3*, plasmodesmata-located proteins 1/2/3; *RHD3*, root hair defective 3; *RIM1*, rice dwarf virus multiplication 1, related to an *Arabidopsis* NAC domain protein (ANAC028); *SAMS*, S-adenosyl-L-methionine synthetase.*

## CRISPR/Cas System-Mediated Resistance to Plant Viruses by Targeting the Viral Genome

### CRISPR/Cas System-Mediated Resistance to Plant DNA Viruses

Geminiviridae and Caulimoviridae are two major destructive plant DNA virus families that contain 485 species with single-stranded DNA (ssDNA) genome and 85 species with double-stranded DNA (dsDNA) genome, respectively, see text foot note 1. Multiple independent works have aimed to directly target and destroy the genomic DNA of geminiviruses or caulimoviruses by plant gene editing technologies. Before the emergence of the CRISPR/Cas systems, the zinc finger nucleases (ZFNs) and transcription activator-like effector nucleases (TALENs) have been applied to manipulate the host and viral DNAs in plants. ZFN- and TALEN-mediated resistance to several geminiviruses, including tomato yellow leaf curl China virus (TYLCCNV) and tobacco curly shoot virus (TbCSV), by targeting the viral genomic replication-associated region has been reported (Chen et al., 2014; Cheng et al., 2015). Compared to ZFN- and TALEN-mediated DNA editing technologies, CRISPR/Cas systems have more advantages in manipulation, so they have rapidly become more popular and promising in antiviral engineering in plants.

CRISPR/Cas9 constructs with single guide RNAs (sgRNAs) targeting the viral replication-associated region, or intergenic region (IR), have exhibited effective DNA interference and conferred viral resistance against beet severe curly top virus (BSCTV), cotton leaf curl Multan virus (CLCuMuV), and bean yellow dwarf virus (BeYDV) in transgenic *Nicotiana benthamiana* or *Arabidopsis thaliana* plants, respectively (Baltes et al., 2015; Ji et al., 2015; Yin et al., 2019). Targeting the tomato yellow leaf curl virus (TYLCV) genome with Cas9–single guide RNA at the sequences encoding the coat protein (CP) or replicase (Rep) resulted in efficient virus interference, as evidenced by the low accumulation of the TYLCV DNA genome in the transgenic tomato and *N. benthamiana* plants (Tashkandi et al., 2018). The sgRNAs targeting the stem-loop sequence compared to the sgRNAs targeting the viral CP region and the replication-associated region within the IR displayed a more effective interference of several geminiviruses including the monopartite geminivirus, cotton leaf curl Kokhran virus (CLCuKoV), the bipartite geminivirus, Merremia mosaic virus (MeMV), and different severe and mild strains of TYLCV geminiviruses (Ali et al., 2016). These findings suggest that CRISPR/Cas9-induced variants in open reading frames (ORFs) but not the IR of geminiviruses are capable of replication and systemic movement, thereby evading the CRISPR/Cas9 machinery. The use of this system by a single sgRNA targeting the conserved stem-loop sequence of the origin of replication in the TYLCV intergenic region was also able to render plants broad-spectrum resistance to other geminiviruses, including a monopartite geminivirus, beet curly top virus (BCTV), and a bipartite geminivirus, MeMV (Ali et al., 2015).

With the fast development of CRISPR/Cas9 system-based technologies, more and more successful examples show the promising resistance to plant DNA viruses. For example, a CRISPR/Cas9 system duplexed with sgRNAs targeting the movement protein (MP) or CP region established resistance to wheat dwarf virus (WDV) or banana streak virus (BSV), respectively (Kis et al., 2019; Tripathi et al., 2019). This approach shall also provide new avenues for engineering cotton against cotton leaf curl disease (CLCuD), mediated by cotton leaf curl virus (CLCuV), a key limiting factor for cotton productivity worldwide (Uniyal et al., 2019). The initial success of transgenic plants engineered by CRISPR/Cas9 systems resistant to geminiviruses points out the potential of a similar strategy against other severe ssDNA viruses, like banana bunchy top virus (BBTV), which causes devastating damage to the whole banana industry in Southern China (Rao et al., 2017).

In addition, CRISPR/Cas9-mediated immunity has been recently utilized to defend against plant dsDNA viruses. *A. thaliana* transgenic plants consistently expressing both Cas9 protein and sgRNAs targeting the CP region of cauliflower mosaic virus (CaMV) in the Caulimoviridae family conferred effective resistance to this species (Liu et al., 2018).

### CRISPR/Cas System-Mediated Resistance to Plant RNA Viruses

Recently, some RNA targeting and editing CRISPR-associated proteins were found, such as Cas9 derived from *Francisella novicida* (FnCas9) and Cas13a (formerly called C2c2) from *Leptotrichia shahii* (LshCas13a) (Price et al., 2015; Shmakov et al., 2015). Among them, LshCas13a is the first Cas13 ortholog to be harnessed for programmable RNA-targeting activities, which expanded the application of CRISPR/Cas systems from DNA to RNA (Abudayyeh et al., 2016). The LshCas13a protein combined with the protospacers can be bioengineered to knock down specific mRNAs in bacteria (Abudayyeh et al., 2017), which offered virologists an alternative CRISPR/Cas system to fight against pathogenic RNA viruses. Therefore, RNA viruses can be directly interfered with by targeting their genome through a CRISPR/Cas system with these new types of Cas effector proteins. Programmable FnCas9 protein and an RNA-targeting gRNA (rgRNA) could target a single-stranded RNA (ssRNA) virus, hepatitis C virus (HCV) in mammalian cells, resulting in the inhibition of viral protein production (Price et al., 2015). A plant codon-optimized version of FnCas9 paired with rgRNA targeting plant ssRNA viruses was also employed to gain viral resistance to cucumber mosaic virus (CMV) and tobacco mosaic virus (TMV) in transgenic *N. benthamiana* and *A. thaliana* plants, respectively (Zhang T. et al., 2018).

Another RNA-targeting CRISPR/Cas system using the class 2 type VI-A CRISPR/Cas effector, LshCas13a, has been characterized and programmed to cleave ssRNA viruses in plants. As expected, LshCas13a with a sgRNA targeting viral RNA sequences exhibited rapid and effective RNA interference to turnip mosaic virus (TuMV), wherein sgRNA was driven by a pea early browning virus (PEBV) promoter in a tobacco rattle virus (TRV)-based vector in LshCas13a transgenic *N. benthamiana* or *A. thaliana* plants, respectively (Aman et al., 2018a,b). Similarly, resistance to TMV, rice stripe mosaic virus (RSMV), and Southern rice black-streaked dwarf virus (SRBSDV) in transgenic tobacco and rice harboring LshCas13a specifically targeting viral RNA was also confirmed using this system (Zhang et al., 2019a). By targeting the P3-, NIb-, or CP-coding sequences in the potato virus Y (PVY) genomic region, this CRISPR/Cas13a system also showed its effectiveness in interfering with and inhibiting PVY infection (Zhan et al., 2019).

Furthermore, a new protein of Cas13 from *Ruminococcus flavefaciens* was characterized and classified into the type VI-D effector, named Cas13d (CasRx). This Cas13d effector with two higher eukaryotic and prokaryotic nucleotide-binding domains (HEPN) that dictate CRISPR RNA maturation and target cleavage also possesses RNase activity to target ssRNA (Konermann et al., 2018). Researchers found that Cas13d showed great advantages over Cas13a, Cas13b, or other Cas13 variants when it was used to interfere with TuMV infection by targeting the GFP, CP, or HC-Pro region in the TuMV-GFP genome (Mahas et al., 2019). Similarly, the Cas13d-mediated RNA interference system was also applied in mammalian cells to combat the novel coronavirus SARS-CoV-2 as well as influenza (Abbott et al., 2020).

## CRISPR/Cas System-Mediated Resistance to Plant Viruses by Targeting Host Factors

Plant viruses recruit their host cellular translation factors not only to translate their viral RNAs but also to facilitate their other infection processes, so the translation-related host factors were initially identified as pro-viral factors. Later, more and more pro-viral host factors, including translation/replication/movement/metabolism-related genes, have been identified and characterized and exploited to control viral diseases in plants. Recessive resistance is conferred by a recessive gene mutation that encodes a host factor critical for viral infection/life cycle or encodes a negative regulator of plant defense responses (Hashimoto et al., 2016). Therefore, it is ideal for obtaining a targeted recessive gene mutant by CRISPR/Cas systems. Eukaryotic translation initiation factors eIF4E, eIF4G, and their isoforms, eIF(iso)4E and eIF(iso)4G, are the most widely exploited recessive resistance genes required for plant RNA virus–protein translation processes in several crop species, and they are effective in defending against a subset of viral species (Lellis et al., 2002; Robaglia and Caranta, 2006; Wang and Krishnaswamy, 2012; Sanfaçon, 2015; Wang, 2015; Hashimoto et al., 2016). It has been reported that CRISPR/Cas9-edited *eIF4E-*knockout cucumber showed complete resistance to zucchini yellow mosaic virus (ZYMV), papaya ringspot mosaic virus-W (PRSV-W), and cucumber vein yellowing virus (CVYV) (Chandrasekaran et al., 2016). Also, a novel allele of rice, *eIF4G*, generated by CRISPR/Cas9-targeted mutagenesis showed resistance to rice tungro spherical virus (RTSV) (Macovei et al., 2018). The induced mutation in CRISPR/Cas9-mediated *eIF(iso)4E* in cassava and *A. thaliana* conferred full resistance to cassava brown streak virus (CBSV) or TuMV-GFP, respectively (Pyott et al., 2016; Gomez et al., 2019). Cassava encodes some alternative eIF4E-like proteins: novel cap-binding protein-1 (nCBP-1) and nCBP-2. When CRISPR/SpCas9 was used to target nCBP-1 or nCBP-2 in cassava plants, the mutant plants displayed successful resistance to CBSV (Gomez et al., 2019). The newly emerged CRISPR–Cas9n–cytidine base editor was also applied in antivirus engineering, which could convert the *Arabidopsis eIF4E1* susceptibility allele into a resistance allele by introducing the N176K mutation to generate clover yellow vein virus (ClYVV)-resistant plants (Bastet et al., 2019). Recently, a soybean PBS1 decoy protein modified to contain a cleavage site for the soybean mosaic virus (SMV) NIa protease triggers cell death in soybean protoplasts, indicating that this CRISPR/Cas system can also be utilized to generate soybean resources resistant to SMV (Helm et al., 2019).

However, the current establishment of efficient, recessive resistance-type antiviral strategies was mainly dependent on eIF4s and their homologs against potyviruses and some related plant viruses. Therefore, more host susceptibility genes need to be identified and applied to obtain effective genetic resources against many other economically important plant viruses.

## The Promising Host Susceptibility Genes for Antiviral Engineering in Plants

Although many dominant resistance genes against plant viruses have been identified, they are often genetically linked to undesired traits, including low yield, poor flavor, small size, and even developmental abnormities. Therefore, these resistance genes would not be valuable in antivirus engineering. Similarly, many host susceptibility genes involved in viral infection also affect plant viability. For example, the *Arabidopsis ssi2* mutant, which accumulates high levels of the plant defense hormone salicylic acid (SA), confers resistance to CMV, but shows an abnormal growth phenotype (Sekine et al., 2004).

Here, we list some promising potential host factors to explore for antiviral breeding whose mutation or deficiency would not perturb plant growth or development, but can still confer resistance to many plant viruses mainly based on these reports (Lozano-Durán et al., 2011; Wang, 2015; Hashimoto et al., 2016; Garcia-Ruiz, 2018; Li and Wang, 2019; Li et al., 2019; Mäkinen, 2020; Table 1). Most of these host factors are related to viruses of the Geminiviridae family (nine genera, 485 species) and the Potyviridae family (12 genera, 228 species), which constitute almost half of the plant viruses described to date see text foot note 1 and cause devastating diseases in economically important crops. Based on the functions of these host factors, we classified them into four groups as follows:

(1) The negative regulators of plant defenses, such as rgs-CaM that inhibited RNA silencing through repressing the transcription level of RDR6 and degrading the SGS3 protein *via* autophagy (Li et al., 2018), and homeodomain leucine zipper protein 1 (HAT1) and its related genes HAT2 and HAT3, whose mutation conferred loss-of-susceptibility to CMV infection by accumulating high levels of SA and jasmonic acid (JA) (Zou et al., 2016).

(2) The susceptible factors involved in viral replication/translation/movement or other life cycles, such as tobamovirus multiplication 1 (TOM1) and its homologs involved in the replication of tobamoviruses (Yamanaka et al., 2000), eEF1A and eEF4s involved in the translation of potyviruses, and PCaP1 (plasma membrane-associated cation-binding protein-1) and Sec24a(a COPII coatomer) involved in the movement of potyviruses (Wang, 2015).

(3) The host factors participating in the modification of viral proteins to achieve their effective function in the infection, such as shaggy-related protein kinases (SK4-1, NsAK, etc.) which could interact with and phosphorylate geminiviral C4 proteins, and are required to trigger disease symptoms (Lozano-Durán et al., 2011). The CP protein of potato virus A (PVA) is phosphorylated by cellular CK2, and the phosphorylated CP interacts with ubiquitin ligase CP-interacting protein (CPIP) and HSP90, which is required for PVA replication and CP accumulation (Lõhmus et al., 2017).

(4) The factors involved in the cellular processes beneficial for virus behaviors, such as phenylpropanoid metabolism factor (4-coumarate:CoA ligase1, 4CL1) or secondary cell wall synthesis factor (Bearskin2B, BRN2), whose silencing in phloem tissue delays geminivirus infection (Lozano-Durán et al., 2011).

The above recessive genetic resources can be obtained by a regular CRISPR/Cas9 system, and the corresponding transgene-free resources can also be further generated by genetic crossing to remove the Cas9 protein out. Different strategies to deliver the constructs expressing Cas proteins or sgRNAs to the plant have been reported and improved. The use of viral vectors to deliver sgRNAs and/or Cas proteins vastly enhanced the efficiency of this system due to the rapid and robust amplification/expression of sgRNAs and/or Cas proteins during virus infection. In addition, the virus vector-based CRISPR/Cas9 system was able to produce plants with the desired traits without the involvement of laborious and time-intensive tissue culture practices. For example, the CRISPR/Cas9 system based on DNA viruses (BeYDV, WDV, and cabbage leaf curl virus) and RNA viruses (TRV, PEBV, TMV, beet necrotic yellow vein virus, and barley stripe mosaic virus) have demonstrated efficient gene targeting frequencies in model plants (*N. benthamiana* and *A. thaliana*) and crops (potato, tomato, rice, wheat, and maize) (Cody et al., 2017; Zaidi and Mansoor, 2017; Hameed et al., 2018; Hu et al., 2019; Jiang et al., 2019). In order to decrease the off-target effects of antiviral strategies mediated by the CRISPR/Cas9 system in plants, (Ji et al., 2018) developed two virus-induced gene editing vectors, in which the pV86 and pC86 promoters of BSCTV were *trans*-activated by co-infecting BSCTV. These BSCTV-inducible vectors were spatially and temporally responsive and inhibited BSCTV accumulation in both transient (*N. benthamiana*) and transgenic (*A. thaliana*) assays without off-target defect. A recent report showed that the sonchus yellow net virus (SYNV)-delivered transgene-free CRISPR/Cas9 system could generate the genetic resources only by one step (Ma et al., 2020), which would save time and labor in breeding procedures. In addition, SYNV is a negative-stranded RNA virus that cannot be integrated into the plant genome or inherited into the next seedlings. Therefore, it is promising to deliver some other CRISPR/Cas editing agents by this system, such as base editors and primer editors, in order to achieve more specific and accurate gene editing results in the future (Anzalone et al., 2020). However, the SYNV-delivered transgene-free CRISPR/Cas9 system only works in *N. benthamiana* plants, which would limit its application in crops. Therefore, more convenient and optimized virus-mediated CRISPR/Cas9 systems need to be explored and applied in antiviral engineering.

## The Potential Risk of CRISPR/Cas Systems in Antiviral Engineering

The CRISPR/Cas systems provide many valuable tools and new thoughts in creating gene loss-of-function and gain-of-function mutants of plants, especially for economic crops. In the plant virus-combating arsenal, the interaction and battle between host plants and viruses never end. When plant virologists are determined to control the dissemination of plant DNA virus by using CRISPR/Cas9 technology to interfere with and destroy viral genome DNA, some accompanying challenges also come out.

The mutation or recombination of endogenous genes would lead to inactivate their function, while the sequence variations of plant viruses generated by genome editing technologies would probably facilitate viral evolution. It has been shown that mutation, recombination, and reassortment are the main driving forces of plant RNA viruses, and the first two are that of plant DNA viruses. Mehta et al. found transgenic cassava plants with the expressions of both Cas9 and a sgRNA targeting the overlapping regions of AC2 and AC3 of African cassava mosaic virus (ACMV) failing to confer any resistance to ACMV infection. The depth analysis of the full-length viral genomes in transgenic plants revealed about 33–48% of inserted geminiviruses. Although these new variants of ACMV generated by CRISPR/Cas9 could not proliferate themselves alone, they are able to rely on wild-type ACMV to accumulate in *N. benthamiana* (Mehta et al., 2019). Ali et al. and Liu et al. also showed the CRISPR/Cas9-mediated targeting of viral ORFs that generated more viral variants, which may result in different levels of viral escape events, raising the possibility that this system has the potential to create and allow the dissemination of mutant viruses from plants even with multiple gRNAs (Ali et al., 2016; Liu et al., 2018). However, the combinations of two single gRNAs, especially those far from each other, would substantially delay resistance breakdown compared to a single sgRNA (Liu et al., 2018). Also, there exist off-target effects when the CRISPR/Cas9 system was applied in *A. thaliana* plants, indicating that this system would possibly entail unpredictable risks (Zhang Q. et al., 2018). In addition, the presence of folded dsRNA domains in sgRNAs could generate small interfering RNAs (siRNAs), which could reduce the abundance of sgRNAs, a possibility that should be considered in the future application of the CRISPR/Cas9 system.

Therefore, the cases and risks that viruses escaped from the CRISPR/Cas system-mediated antiviral immunity in plants warn that the CRISPR/Cas system may be considered as a double-edged sword in antiviral engineering. Although it can target, destroy, or interfere with the viral genome to inhibit viral infection, it raises an important issue that the new viral variants or species may be generated as genome editing by-products, indicating that it would speed up the virus evolution or that the obtained transgenic crops may lose their specific resistance to these viruses.

## Conclusion and Future Perspectives

Molecular breeding has played a pivotal role in the prevention and control of plant viruses causing diseases, and the emergence and development of CRISPR/Cas technologies would speed up the generation and obtainment of new resistance resources. With the development and understanding of CRISPR/Cas systems and the exploration and function identification of recessive resistance genes, the resistance materials with the mutation or deficiency in host susceptibility factors generated by CRISPR/Cas systems would better benefit viral prevention and control in the field. Compared to targeting, destroying, or interfering with viral genomes, which might increase the risks of viral evolution, the editing of recessive resistance genes would have a promising perspective in engineering-resistant plants against plant viruses. Additionally, host susceptibility genes for insect vectors can be important targets by CRISPR/Cas systems to prevent the viral spread and dissemination. Of note is that it is also useful for antiviral engineering by utilizing newly developed CRISPR/Cas systems. For instance, the transcriptional activation of many plant-dominant resistance genes by CRISPR-Act2.0 would enhance the plant immunity to confer a broad range of resistance to different kinds of pathogens (Lowder et al., 2018), and the primer editor is also an interesting alternative applied into the antiviral engineering (Anzalone et al., 2019). The multiplexed CRISPR technologies, in which numerous gRNAs or Cas proteins are expressed at once, have vastly enhanced the scope and efficiency of genetic editing and transcriptional regulation (McCarty et al., 2020). However, the application of these multiplexed CRISPR technologies into the field to control plant viruses is still on the way, mainly because of the complexity of experimental manipulation, the limitations of the genetic transformation of several simultaneous Cas proteins into one plant, and the lack of validated and effective endogenous targets against different viruses, among other things (McCarty et al., 2020).

## Author Contributions

FL, HZ, and XZ conceived the project. YC, FL, and XZ wrote the manuscript with contributions from all authors.

## Conflict of Interest

The authors declare that the research was conducted in the absence of any commercial or financial relationships that could be construed as a potential conflict of interest.

## Footnotes

1 https://talk.ictvonline.org/taxonomy/
